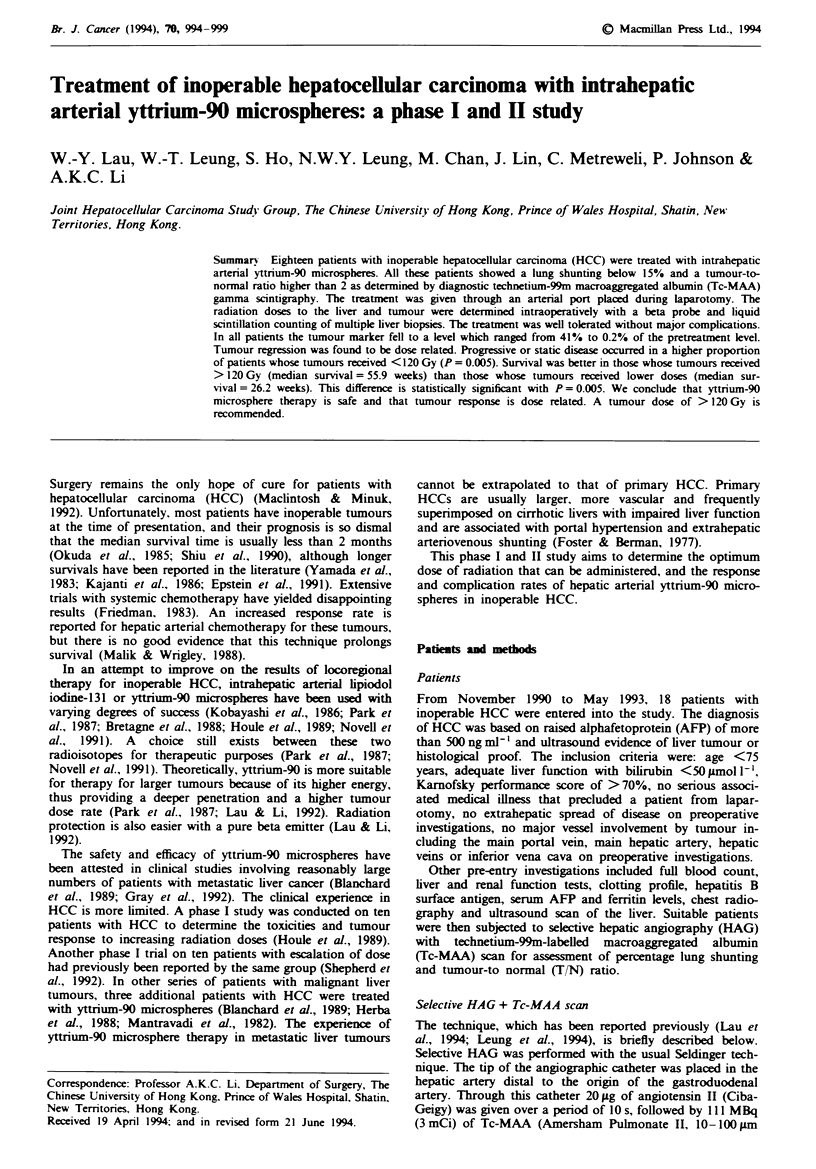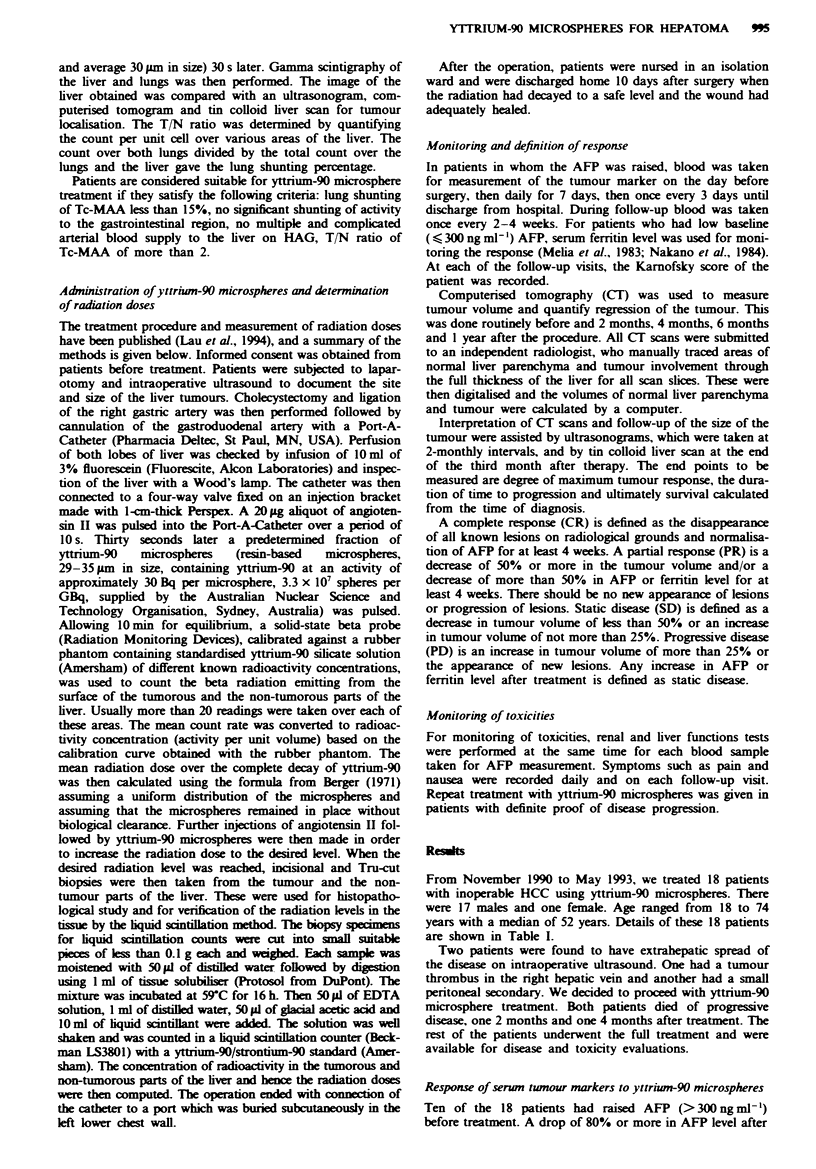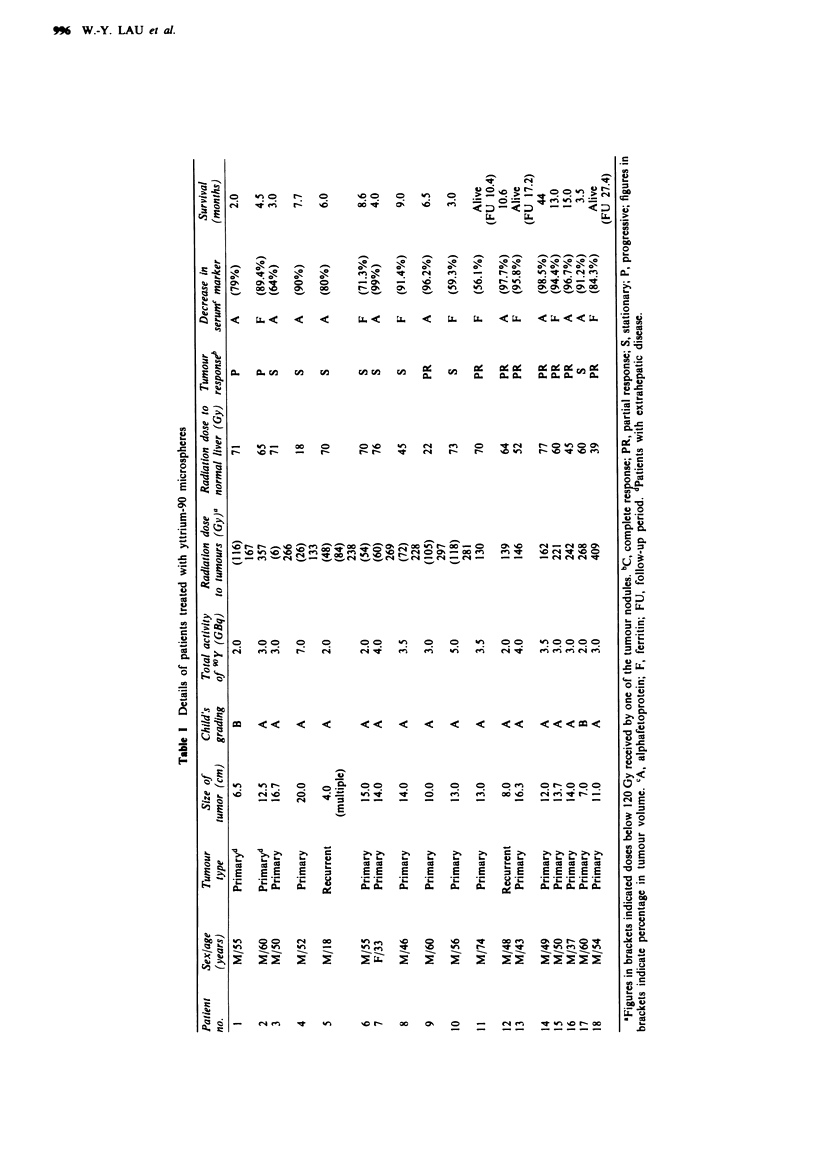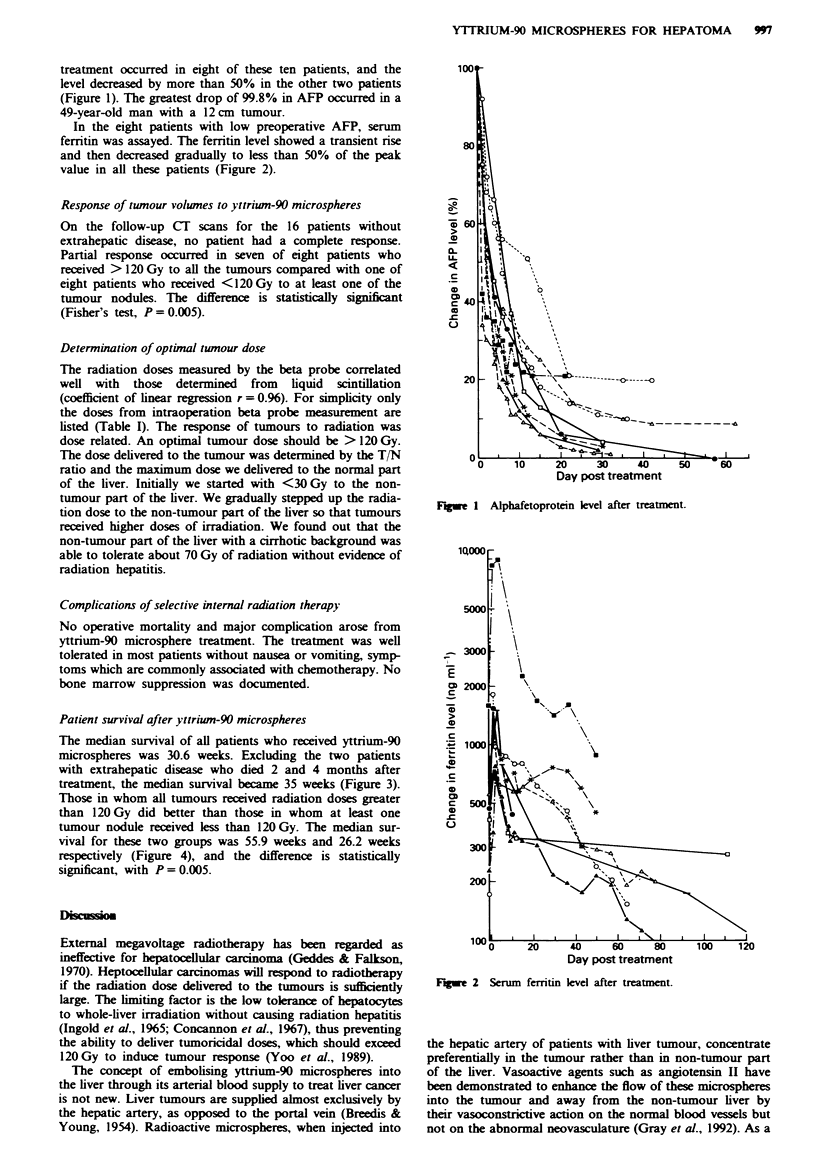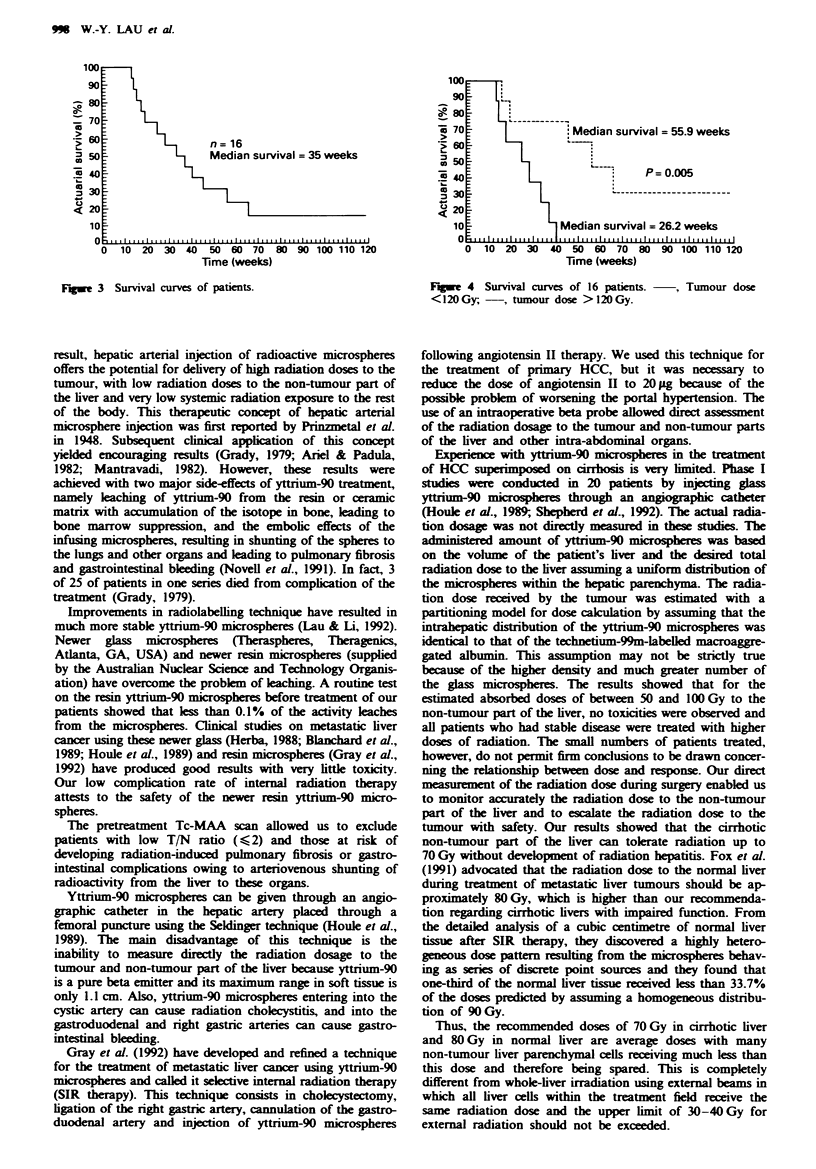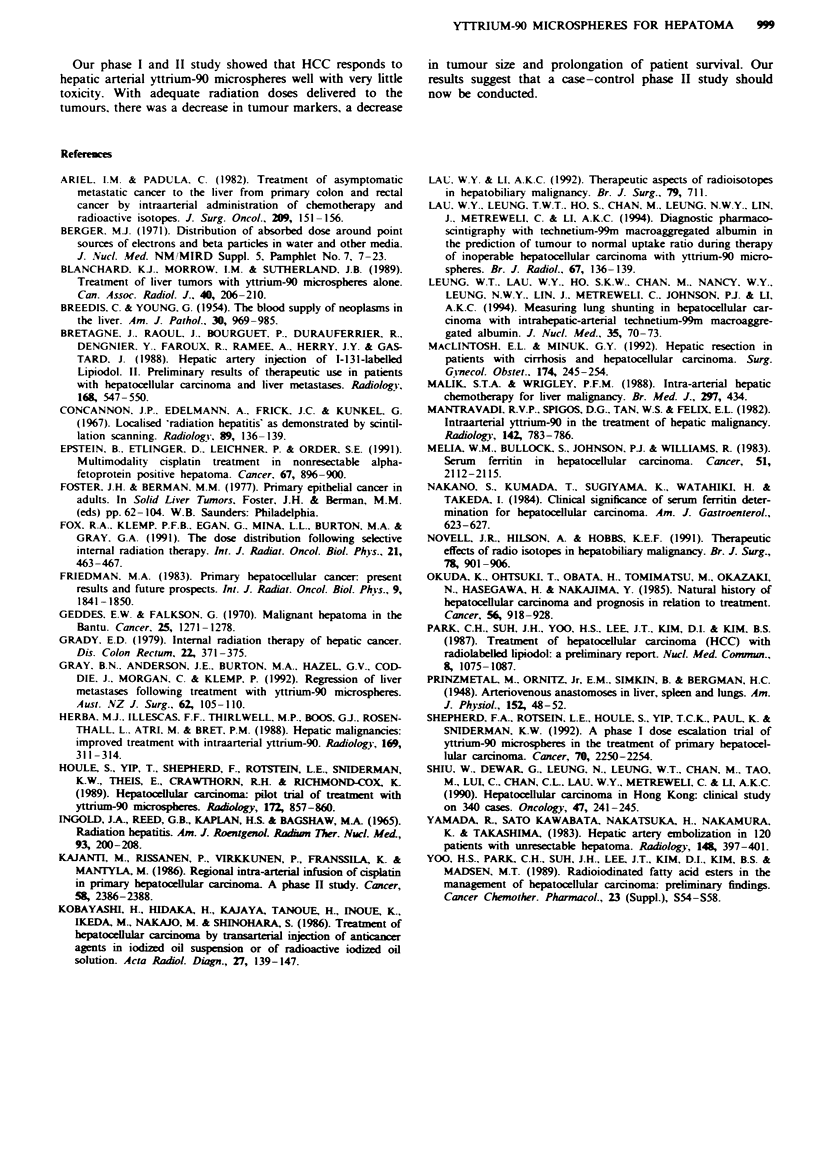# Treatment of inoperable hepatocellular carcinoma with intrahepatic arterial yttrium-90 microspheres: a phase I and II study.

**DOI:** 10.1038/bjc.1994.436

**Published:** 1994-11

**Authors:** W. Y. Lau, W. T. Leung, S. Ho, N. W. Leung, M. Chan, J. Lin, C. Metreweli, P. Johnson, A. K. Li

**Affiliations:** Chinese University of Hong Kong, Prince of Wales Hospital, Shatin, New Territories.

## Abstract

Eighteen patients with inoperable hepatocellular carcinoma (HCC) were treated with intrahepatic arterial yttrium-90 microspheres. All these patients showed a lung shunting below 15% and a tumour-to-normal ratio higher than 2 as determined by diagnostic technetium-99m macroaggregated albumin (Tc-MAA) gamma scintigraphy. The treatment was given through an arterial port placed during laparotomy. The radiation doses to the liver and tumour were determined intraoperatively with a beta probe and liquid scintillation counting of multiple liver biopsies. The treatment was well tolerated without major complications. In all patients the tumour marker fell to a level which ranged from 41% to 0.2% of the pretreatment level. Tumour regression was found to be dose related. Progressive or static disease occurred in a higher proportion of patients whose tumours received < 120 Gy (P = 0.005). Survival was better in those whose tumours received > 120 Gy (median survival = 55.9 weeks) than those whose tumours received lower doses (median survival = 26.2 weeks). This difference is statistically significant with P = 0.005. We conclude that yttrium-90 microsphere therapy is safe and that tumour response is dose related. A tumour dose of > 120 Gy is recommended.


Br. J. Cancer (1994), 70, 994–999 © Macmillan Press Ltd., 1994

### Treatment of inoperable hepatocellular carcinoma with intrahepatic arterial yttrium-90 microspheres: a phase I and II study

W.-Y. Lau, W.-T. Leung, S. Ho, N.W.Y. Leung, M. Chan, J. Lin, C. Metreweli, P. Johnson & A.K.C. Li

*Joint Hepatocellular Carcinoma Study Group, The Chinese University of Hong Kong, Prince of Wales Hospital, Shatin, New Territories, Hong Kong.*

**Summary** Eighteen patients with inoperable hepatocellular carcinoma (HCC) were treated with intrahepatic arterial yttrium-90 microspheres. All these patients showed a lung shunting below 15% and a tumour-to-normal ratio higher than 2 as determined by diagnostic technetium-99m macroaggregated albumin (Tc-MAA) gamma scintigraphy. The treatment was given through an arterial port placed during laparotomy. The radiation doses to the liver and tumour were determined intraoperatively with a beta probe and liquid scintillation counting of multiple liver biopsies. The treatment was well tolerated without major complications. In all patients the tumour marker fell to a level which ranged from 41% to 0.2% of the pretreatment level. Tumour regression was found to be dose related. Progressive or static disease occurred in a higher proportion of patients whose tumours received <120 Gy ($P = 0.005$). Survival was better in those whose tumours received >120 Gy (median survival = 55.9 weeks) than those whose tumours received lower doses (median survival = 26.2 weeks). This difference is statistically significant with $P = 0.005$. We conclude that yttrium-90 microsphere therapy is safe and that tumour response is dose related. A tumour dose of >120 Gy is recommended.

Surgery remains the only hope of cure for patients with hepatocellular carcinoma (HCC) (MacIintosh & Minuk, 1992). Unfortunately, most patients have inoperable tumours at the time of presentation, and their prognosis is so dismal that the median survival time is usually less than 2 months (Okuda *et al.*, 1985; Shiu *et al.*, 1990), although longer survivals have been reported in the literature (Yamada *et al.*, 1983; Kajanti *et al.*, 1986; Epstein *et al.*, 1991). Extensive trials with systemic chemotherapy have yielded disappointing results (Friedman, 1983). An increased response rate is reported for hepatic arterial chemotherapy for these tumours, but there is no good evidence that this technique prolongs survival (Malik & Wrigley, 1988).

In an attempt to improve on the results of locoregional therapy for inoperable HCC, intrahepatic arterial lipiodol iodine-131 or yttrium-90 microspheres have been used with varying degrees of success (Kobayashi *et al.*, 1986; Park *et al.*, 1987; Bretagne *et al.*, 1988; Houle *et al.*, 1989; Novell *et al.*, 1991). A choice still exists between these two radioisotopes for therapeutic purposes (Park *et al.*, 1987; Novell *et al.*, 1991). Theoretically, yttrium-90 is more suitable for therapy for larger tumours because of its higher energy, thus providing a deeper penetration and a higher tumour dose rate (Park *et al.*, 1987; Lau & Li, 1992). Radiation protection is also easier with a pure beta emitter (Lau & Li, 1992).

The safety and efficacy of yttrium-90 microspheres have been attested in clinical studies involving reasonably large numbers of patients with metastatic liver cancer (Blanchard *et al.*, 1989; Gray *et al.*, 1992). The clinical experience in HCC is more limited. A phase I study was conducted on ten patients with HCC to determine the toxicities and tumour response to increasing radiation doses (Houle *et al.*, 1989). Another phase I trial on ten patients with escalation of dose had previously been reported by the same group (Shepherd *et al.*, 1992). In other series of patients with malignant liver tumours, three additional patients with HCC were treated with yttrium-90 microspheres (Blanchard *et al.*, 1989; Herba *et al.*, 1988; Mantravadi *et al.*, 1982). The experience of yttrium-90 microsphere therapy in metastatic liver tumours cannot be extrapolated to that of primary HCC. Primary HCCs are usually larger, more vascular and frequently superimposed on cirrhotic livers with impaired liver function and are associated with portal hypertension and extrahepatic arteriovenous shunting (Foster & Berman, 1977).

This phase I and II study aims to determine the optimum dose of radiation that can be administered, and the response and complication rates of hepatic arterial yttrium-90 microspheres in inoperable HCC.

### Patients and methods

### *Patients*

From November 1990 to May 1993, 18 patients with inoperable HCC were entered into the study. The diagnosis of HCC was based on raised alphafetoprotein (AFP) of more than 500 ng $ml^{-1}$ and ultrasound evidence of liver tumour or histological proof. The inclusion criteria were: age <75 years, adequate liver function with bilirubin <50 $\mu mol\ l^{-1}$, Karnofsky performance score of >70%, no serious associated medical illness that precluded a patient from laparotomy, no extrahepatic spread of disease on preoperative investigations, no major vessel involvement by tumour including the main portal vein, main hepatic artery, hepatic veins or inferior vena cava on preoperative investigations.

Other pre-entry investigations included full blood count, liver and renal function tests, clotting profile, hepatitis B surface antigen, serum AFP and ferritin levels, chest radiography and ultrasound scan of the liver. Suitable patients were then subjected to selective hepatic angiography (HAG) with technetium-99m-labelled macroaggregated albumin (Tc-MAA) scan for assessment of percentage lung shunting and tumour-to normal (T/N) ratio.

### *Selective HAG + Tc-MAA scan*

The technique, which has been reported previously (Lau *et al.*, 1994; Leung *et al.*, 1994), is briefly described below. Selective HAG was performed with the usual Seldinger technique. The tip of the angiographic catheter was placed in the hepatic artery distal to the origin of the gastroduodenal artery. Through this catheter 20 $\mu g$ of angiotensin II (Ciba-Geigy) was given over a period of 10 s, followed by 111 MBq (3 mCi) of Tc-MAA (Amersham Pulmonate II, 10–100 $\mu m$

Correspondence: Professor A.K.C. Li, Department of Surgery, The Chinese University of Hong Kong, Prince of Wales Hospital, Shatin, New Territories, Hong Kong.
Received 19 April 1994; and in revised form 21 June 1994.

and average 30 μm in size) 30 s later. Gamma scintigraphy of the liver and lungs was then performed. The image of the liver obtained was compared with an ultrasonogram, computerised tomogram and tin colloid liver scan for tumour localisation. The T/N ratio was determined by quantifying the count per unit cell over various areas of the liver. The count over both lungs divided by the total count over the lungs and the liver gave the lung shunting percentage.

Patients are considered suitable for yttrium-90 microsphere treatment if they satisfy the following criteria: lung shunting of Tc-MAA less than 15%, no significant shunting of activity to the gastrointestinal region, no multiple and complicated arterial blood supply to the liver on HAG, T/N ratio of Tc-MAA of more than 2.

### *Administration of yttrium-90 microspheres and determination of radiation doses*

The treatment procedure and measurement of radiation doses have been published (Lau *et al.*, 1994), and a summary of the methods is given below. Informed consent was obtained from patients before treatment. Patients were subjected to laparotomy and intraoperative ultrasound to document the site and size of the liver tumours. Cholecystectomy and ligation of the right gastric artery was then performed followed by cannulation of the gastroduodenal artery with a Port-A-Catheter (Pharmacia Deltec, St Paul, MN, USA). Perfusion of both lobes of liver was checked by infusion of 10 ml of 3% fluorescein (Fluorescite, Alcon Laboratories) and inspection of the liver with a Wood's lamp. The catheter was then connected to a four-way valve fixed on an injection bracket made with 1-cm-thick Perspex. A 20 μg aliquot of angiotensin II was pulsed into the Port-A-Catheter over a period of 10 s. Thirty seconds later a predetermined fraction of yttrium-90 microspheres (resin-based microspheres, 29–35 μm in size, containing yttrium-90 at an activity of approximately 30 Bq per microsphere, $3.3 \times 10^7$ spheres per GBq, supplied by the Australian Nuclear Science and Technology Organisation, Sydney, Australia) was pulsed. Allowing 10 min for equilibrium, a solid-state beta probe (Radiation Monitoring Devices), calibrated against a rubber phantom containing standardised yttrium-90 silicate solution (Amersham) of different known radioactivity concentrations, was used to count the beta radiation emitting from the surface of the tumorous and the non-tumorous parts of the liver. Usually more than 20 readings were taken over each of these areas. The mean count rate was converted to radioactivity concentration (activity per unit volume) based on the calibration curve obtained with the rubber phantom. The mean radiation dose over the complete decay of yttrium-90 was then calculated using the formula from Berger (1971) assuming a uniform distribution of the microspheres and assuming that the microspheres remained in place without biological clearance. Further injections of angiotensin II followed by yttrium-90 microspheres were then made in order to increase the radiation dose to the desired level. When the desired radiation level was reached, incisional and Tru-cut biopsies were then taken from the tumour and the non-tumour parts of the liver. These were used for histopathological study and for verification of the radiation levels in the tissue by the liquid scintillation method. The biopsy specimens for liquid scintillation counts were cut into small suitable pieces of less than 0.1 g each and weighed. Each sample was moistened with 50 μl of distilled water followed by digestion using 1 ml of tissue solubiliser (Protosol from DuPont). The mixture was incubated at 59°C for 16 h. Then 50 μl of EDTA solution, 1 ml of distilled water, 50 μl of glacial acetic acid and 10 ml of liquid scintillant were added. The solution was well shaken and was counted in a liquid scintillation counter (Beckman LS3801) with a yttrium-90/strontium-90 standard (Amersham). The concentration of radioactivity in the tumorous and non-tumorous parts of the liver and hence the radiation doses were then computed. The operation ended with connection of the catheter to a port which was buried subcutaneously in the left lower chest wall.

After the operation, patients were nursed in an isolation ward and were discharged home 10 days after surgery when the radiation had decayed to a safe level and the wound had adequately healed.

### *Monitoring and definition of response*

In patients in whom the AFP was raised, blood was taken for measurement of the tumour marker on the day before surgery, then daily for 7 days, then once every 3 days until discharge from hospital. During follow-up blood was taken once every 2–4 weeks. For patients who had low baseline ($\leqslant 300$ ng ml$^{-1}$) AFP, serum ferritin level was used for monitoring the response (Melia *et al.*, 1983; Nakano *et al.*, 1984). At each of the follow-up visits, the Karnofsky score of the patient was recorded.

Computerised tomography (CT) was used to measure tumour volume and quantify regression of the tumour. This was done routinely before and 2 months, 4 months, 6 months and 1 year after the procedure. All CT scans were submitted to an independent radiologist, who manually traced areas of normal liver parenchyma and tumour involvement through the full thickness of the liver for all scan slices. These were then digitalised and the volumes of normal liver parenchyma and tumour were calculated by a computer.

Interpretation of CT scans and follow-up of the size of the tumour were assisted by ultrasonograms, which were taken at 2-monthly intervals, and by tin colloid liver scan at the end of the third month after therapy. The end points to be measured are degree of maximum tumour response, the duration of time to progression and ultimately survival calculated from the time of diagnosis.

A complete response (CR) is defined as the disappearance of all known lesions on radiological grounds and normalisation of AFP for at least 4 weeks. A partial response (PR) is a decrease of 50% or more in the tumour volume and/or a decrease of more than 50% in AFP or ferritin level for at least 4 weeks. There should be no new appearance of lesions or progression of lesions. Static disease (SD) is defined as a decrease in tumour volume of less than 50% or an increase in tumour volume of not more than 25%. Progressive disease (PD) is an increase in tumour volume of more than 25% or the appearance of new lesions. Any increase in AFP or ferritin level after treatment is defined as static disease.

### *Monitoring of toxicities*

For monitoring of toxicities, renal and liver functions tests were performed at the same time for each blood sample taken for AFP measurement. Symptoms such as pain and nausea were recorded daily and on each follow-up visit. Repeat treatment with yttrium-90 microspheres was given in patients with definite proof of disease progression.

### Results

From November 1990 to May 1993, we treated 18 patients with inoperable HCC using yttrium-90 microspheres. There were 17 males and one female. Age ranged from 18 to 74 years with a median of 52 years. Details of these 18 patients are shown in Table I.

Two patients were found to have extrahepatic spread of the disease on intraoperative ultrasound. One had a tumour thrombus in the right hepatic vein and another had a small peritoneal secondary. We decided to proceed with yttrium-90 microsphere treatment. Both patients died of progressive disease, one 2 months and one 4 months after treatment. The rest of the patients underwent the full treatment and were available for disease and toxicity evaluations.

### *Response of serum tumour markers to yttrium-90 microspheres*

Ten of the 18 patients had raised AFP ($>300$ ng ml$^{-1}$) before treatment. A drop of 80% or more in AFP level after

**Table I** Details of patients treated with yttrium-90 microspheres

| Patient no. | Sex/age (years) | Tumour type | Size of tumor (cm) | Child's grading | Total activity of $^{90}Y$ (GBq) | Radiation dose to tumours (Gy)[a] | Radiation dose to normal liver (Gy) | Tumour response[b] | Decrease in serum[c] marker | Survival (months) |
|---|---|---|---|---|---|---|---|---|---|---|
| 1 | M/55 | Primary[d] | 6.5 | B | 2.0 | (116)<br>167 | 71 | P | A (79%) | 2.0 |
| 2 | M/60 | Primary[d] | 12.5 | A | 3.0 | 357 | 65 | P | F (89.4%) | 4.5 |
| 3 | M/50 | Primary | 16.7 | A | 3.0 | (6)<br>266 | 71 | S | A (64%) | 3.0 |
| 4 | M/52 | Primary | 20.0 | A | 7.0 | (26)<br>133 | 18 | S | A (90%) | 7.7 |
| 5 | M/18 | Recurrent | 4.0<br>(multiple) | A | 2.0 | (48)<br>(84)<br>238 | 70 | S | A (80%) | 6.0 |
| 6 | M/55 | Primary | 15.0 | A | 2.0 | (54) | 70 | S | F (71.3%) | 8.6 |
| 7 | F/33 | Primary | 14.0 | A | 4.0 | (60)<br>269 | 76 | S | A (99%) | 4.0 |
| 8 | M/46 | Primary | 14.0 | A | 3.5 | (72)<br>228 | 45 | S | F (91.4%) | 9.0 |
| 9 | M/60 | Primary | 10.0 | A | 3.0 | (105)<br>297 | 22 | PR | A (96.2%) | 6.5 |
| 10 | M/56 | Primary | 13.0 | A | 5.0 | (118)<br>281 | 73 | S | F (59.3%) | 3.0 |
| 11 | M/74 | Primary | 13.0 | A | 3.5 | 130 | 70 | PR | F (56.1%) | Alive<br>(FU 10.4) |
| 12 | M/48 | Recurrent | 8.0 | A | 2.0 | 139 | 64 | PR | A (97.7%) | 10.6 |
| 13 | M/43 | Primary | 16.3 | A | 4.0 | 146 | 52 | PR | F (95.8%) | Alive<br>(FU 17.2) |
| 14 | M/49 | Primary | 12.0 | A | 3.5 | 162 | 77 | PR | A (98.5%) | 44 |
| 15 | M/50 | Primary | 13.7 | A | 3.0 | 221 | 60 | PR | F (94.4%) | 13.0 |
| 16 | M/37 | Primary | 14.0 | A | 3.0 | 242 | 45 | PR | A (96.7%) | 15.0 |
| 17 | M/60 | Primary | 7.0 | B | 2.0 | 268 | 60 | S | A (91.2%) | 3.5 |
| 18 | M/54 | Primary | 11.0 | A | 3.0 | 409 | 39 | PR | F (84.3%) | Alive<br>(FU 27.4) |

[a]Figures in brackets indicated doses below 120 Gy received by one of the tumour nodules. [b]C, complete response; PR, partial response; S, stationary; P, progressive; figures in brackets indicate percentage in tumour volume. [c]A, alphafetoprotein; F, ferritin; FU, follow-up period. [d]Patients with extrahepatic disease.

treatment occurred in eight of these ten patients, and the level decreased by more than 50% in the other two patients (Figure 1). The greatest drop of 99.8% in AFP occurred in a 49-year-old man with a 12 cm tumour.

In the eight patients with low preoperative AFP, serum ferritin was assayed. The ferritin level showed a transient rise and then decreased gradually to less than 50% of the peak value in all these patients (Figure 2).

*Response of tumour volumes to yttrium-90 microspheres*

On the follow-up CT scans for the 16 patients without extrahepatic disease, no patient had a complete response. Partial response occurred in seven of eight patients who received $>120$ Gy to all the tumours compared with one of eight patients who received $<120$ Gy to at least one of the tumour nodules. The difference is statistically significant (Fisher's test, $P = 0.005$).

*Determination of optimal tumour dose*

The radiation doses measured by the beta probe correlated well with those determined from liquid scintillation (coefficient of linear regression $r = 0.96$). For simplicity only the doses from intraoperation beta probe measurement are listed (Table I). The response of tumours to radiation was dose related. An optimal tumour dose should be $>120$ Gy. The dose delivered to the tumour was determined by the T/N ratio and the maximum dose we delivered to the normal part of the liver. Initially we started with $<30$ Gy to the non-tumour part of the liver. We gradually stepped up the radiation dose to the non-tumour part of the liver so that tumours received higher doses of irradiation. We found out that the non-tumour part of the liver with a cirrhotic background was able to tolerate about 70 Gy of radiation without evidence of radiation hepatitis.

*Complications of selective internal radiation therapy*

No operative mortality and major complication arose from yttrium-90 microsphere treatment. The treatment was well tolerated in most patients without nausea or vomiting, symptoms which are commonly associated with chemotherapy. No bone marrow suppression was documented.

*Patient survival after yttrium-90 microspheres*

The median survival of all patients who received yttrium-90 microspheres was 30.6 weeks. Excluding the two patients with extrahepatic disease who died 2 and 4 months after treatment, the median survival became 35 weeks (Figure 3). Those in whom all tumours received radiation doses greater than 120 Gy did better than those in whom at least one tumour nodule received less than 120 Gy. The median survival for these two groups was 55.9 weeks and 26.2 weeks respectively (Figure 4), and the difference is statistically significant, with $P = 0.005$.

### Discussion

External megavoltage radiotherapy has been regarded as ineffective for hepatocellular carcinoma (Geddes & Falkson, 1970). Heptocellular carcinomas will respond to radiotherapy if the radiation dose delivered to the tumours is sufficiently large. The limiting factor is the low tolerance of hepatocytes to whole-liver irradiation without causing radiation hepatitis (Ingold *et al.*, 1965; Concannon *et al.*, 1967), thus preventing the ability to deliver tumoricidal doses, which should exceed 120 Gy to induce tumour response (Yoo *et al.*, 1989).

The concept of embolising yttrium-90 microspheres into the liver through its arterial blood supply to treat liver cancer is not new. Liver tumours are supplied almost exclusively by the hepatic artery, as opposed to the portal vein (Breedis & Young, 1954). Radioactive microspheres, when injected into the hepatic artery of patients with liver tumour, concentrate preferentially in the tumour rather than in non-tumour part of the liver. Vasoactive agents such as angiotensin II have been demonstrated to enhance the flow of these microspheres into the tumour and away from the non-tumour liver by their vasoconstrictive action on the normal blood vessels but not on the abnormal neovasculature (Gray *et al.*, 1992). As a

Figure 1 Alphafetoprotein level after treatment.

Figure 2 Serum ferritin level after treatment.

**Figure 3** Survival curves of patients.

**Figure 4** Survival curves of 16 patients. ——, Tumour dose <120 Gy; ---, tumour dose >120 Gy.

result, hepatic arterial injection of radioactive microspheres offers the potential for delivery of high radiation doses to the tumour, with low radiation doses to the non-tumour part of the liver and very low systemic radiation exposure to the rest of the body. This therapeutic concept of hepatic arterial microsphere injection was first reported by Prinzmetal *et al.* in 1948. Subsequent clinical application of this concept yielded encouraging results (Grady, 1979; Ariel & Padula, 1982; Mantravadi, 1982). However, these results were achieved with two major side-effects of yttrium-90 treatment, namely leaching of yttrium-90 from the resin or ceramic matrix with accumulation of the isotope in bone, leading to bone marrow suppression, and the embolic effects of the infusing microspheres, resulting in shunting of the spheres to the lungs and other organs and leading to pulmonary fibrosis and gastrointestinal bleeding (Novell *et al.*, 1991). In fact, 3 of 25 of patients in one series died from complication of the treatment (Grady, 1979).

Improvements in radiolabelling technique have resulted in much more stable yttrium-90 microspheres (Lau & Li, 1992). Newer glass microspheres (Theraspheres, Theragenics, Atlanta, GA, USA) and newer resin microspheres (supplied by the Australian Nuclear Science and Technology Organisation) have overcome the problem of leaching. A routine test on the resin yttrium-90 microspheres before treatment of our patients showed that less than 0.1% of the activity leaches from the microspheres. Clinical studies on metastatic liver cancer using these newer glass (Herba, 1988; Blanchard *et al.*, 1989; Houle *et al.*, 1989) and resin microspheres (Gray *et al.*, 1992) have produced good results with very little toxicity. Our low complication rate of internal radiation therapy attests to the safety of the newer resin yttrium-90 microspheres.

The pretreatment Tc-MAA scan allowed us to exclude patients with low T/N ratio (⩽2) and those at risk of developing radiation-induced pulmonary fibrosis or gastrointestinal complications owing to arteriovenous shunting of radioactivity from the liver to these organs.

Yttrium-90 microspheres can be given through an angiographic catheter in the hepatic artery placed through a femoral puncture using the Seldinger technique (Houle *et al.*, 1989). The main disadvantage of this technique is the inability to measure directly the radiation dosage to the tumour and non-tumour part of the liver because yttrium-90 is a pure beta emitter and its maximum range in soft tissue is only 1.1 cm. Also, yttrium-90 microspheres entering into the cystic artery can cause radiation cholecystitis, and into the gastroduodenal and right gastric arteries can cause gastrointestinal bleeding.

Gray *et al.* (1992) have developed and refined a technique for the treatment of metastatic liver cancer using yttrium-90 microspheres and called it selective internal radiation therapy (SIR therapy). This technique consists in cholecystectomy, ligation of the right gastric artery, cannulation of the gastroduodenal artery and injection of yttrium-90 microspheres following angiotensin II therapy. We used this technique for the treatment of primary HCC, but it was necessary to reduce the dose of angiotensin II to 20 μg because of the possible problem of worsening the portal hypertension. The use of an intraoperative beta probe allowed direct assessment of the radiation dosage to the tumour and non-tumour parts of the liver and other intra-abdominal organs.

Experience with yttrium-90 microspheres in the treatment of HCC superimposed on cirrhosis is very limited. Phase I studies were conducted in 20 patients by injecting glass yttrium-90 microspheres through an angiographic catheter (Houle *et al.*, 1989; Shepherd *et al.*, 1992). The actual radiation dosage was not directly measured in these studies. The administered amount of yttrium-90 microspheres was based on the volume of the patient's liver and the desired total radiation dose to the liver assuming a uniform distribution of the microspheres within the hepatic parenchyma. The radiation dose received by the tumour was estimated with a partitioning model for dose calculation by assuming that the intrahepatic distribution of the yttrium-90 microspheres was identical to that of the technetium-99m-labelled macroaggregated albumin. This assumption may not be strictly true because of the higher density and much greater number of the glass microspheres. The results showed that for the estimated absorbed doses of between 50 and 100 Gy to the non-tumour part of the liver, no toxicities were observed and all patients who had stable disease were treated with higher doses of radiation. The small numbers of patients treated, however, do not permit firm conclusions to be drawn concerning the relationship between dose and response. Our direct measurement of the radiation dose during surgery enabled us to monitor accurately the radiation dose to the non-tumour part of the liver and to escalate the radiation dose to the tumour with safety. Our results showed that the cirrhotic non-tumour part of the liver can tolerate radiation up to 70 Gy without development of radiation hepatitis. Fox *et al.* (1991) advocated that the radiation dose to the normal liver during treatment of metastatic liver tumours should be approximately 80 Gy, which is higher than our recommendation regarding cirrhotic livers with impaired function. From the detailed analysis of a cubic centimetre of normal liver tissue after SIR therapy, they discovered a highly heterogeneous dose pattern resulting from the microspheres behaving as series of discrete point sources and they found that one-third of the normal liver tissue received less than 33.7% of the doses predicted by assuming a homogeneous distribution of 90 Gy.

Thus, the recommended doses of 70 Gy in cirrhotic liver and 80 Gy in normal liver are average doses with many non-tumour liver parenchymal cells receiving much less than this dose and therefore being spared. This is completely different from whole-liver irradiation using external beams in which all liver cells within the treatment field receive the same radiation dose and the upper limit of 30–40 Gy for external radiation should not be exceeded.

Our phase I and II study showed that HCC responds to hepatic arterial yttrium-90 microspheres well with very little toxicity. With adequate radiation doses delivered to the tumours, there was a decrease in tumour markers, a decrease in tumour size and prolongation of patient survival. Our results suggest that a case-control phase II study should now be conducted.

### References

ARIEL, I.M. & PADULA, C. (1982). Treatment of asymptomatic metastatic cancer to the liver from primary colon and rectal cancer by intraarterial administration of chemotherapy and radioactive isotopes. *J. Surg. Oncol.*, **209**, 151-156.

BERGER, M.J. (1971). Distribution of absorbed dose around point sources of electrons and beta particles in water and other media. *J. Nucl. Med.* NM/MIRD Suppl. 5, Pamphlet No. 7, 7-23.

BLANCHARD, K.J., MORROW, I.M. & SUTHERLAND, J.B. (1989). Treatment of liver tumors with yttrium-90 microspheres alone. *Can. Assoc. Radiol. J.*, **40**, 206-210.

BREEDIS, C. & YOUNG, G. (1954). The blood supply of neoplasms in the liver. *Am. J. Pathol.*, **30**, 969-985.

BRETAGNE, J., RAOUL, J., BOURGUET, P., DURAUFERRIER, R., DENGNIER, Y., FAROUX, R., RAMEE, A., HERRY, J.Y. & GASTARD, J. (1988). Hepatic artery injection of I-131-labelled Lipiodol. II. Preliminary results of therapeutic use in patients with hepatocellular carcinoma and liver metastases. *Radiology*, **168**, 547-550.

CONCANNON, J.P., EDELMANN, A., FRICK, J.C. & KUNKEL, G. (1967). Localised 'radiation hepatitis' as demonstrated by scintillation scanning. *Radiology*, **89**, 136-139.

EPSTEIN, B., ETLINGER, D., LEICHNER, P. & ORDER, S.E. (1991). Multimodality cisplatin treatment in nonresectable alpha-fetoprotein positive hepatoma. *Cancer*, **67**, 896-900.

FOSTER, J.H. & BERMAN, M.M. (1977). Primary epithelial cancer in adults. In *Solid Liver Tumors*, Foster, J.H. & Berman, M.M. (eds) pp. 62-104. W.B. Saunders: Philadelphia.

FOX, R.A., KLEMP, P.F.B., EGAN, G., MINA, L.L., BURTON, M.A. & GRAY, G.A. (1991). The dose distribution following selective internal radiation therapy. *Int. J. Radiat. Oncol. Biol. Phys.*, **21**, 463-467.

FRIEDMAN, M.A. (1983). Primary hepatocellular cancer: present results and future prospects. *Int. J. Radiat. Oncol. Biol. Phys.*, **9**, 1841-1850.

GEDDES, E.W. & FALKSON, G. (1970). Malignant hepatoma in the Bantu. *Cancer*, **25**, 1271-1278.

GRADY, E.D. (1979). Internal radiation therapy of hepatic cancer. *Dis. Colon Rectum*, **22**, 371-375.

GRAY, B.N., ANDERSON, J.E., BURTON, M.A., HAZEL, G.V., CODDIE, J., MORGAN, C. & KLEMP, P. (1992). Regression of liver metastases following treatment with yttrium-90 microspheres. *Aust. NZ J. Surg.*, **62**, 105-110.

HERBA, M.J., ILLESCAS, F.F., THIRLWELL, M.P., BOOS, G.J., ROSENTHALL, L., ATRI, M. & BRET, P.M. (1988). Hepatic malignancies: improved treatment with intraarterial yttrium-90. *Radiology*, **169**, 311-314.

HOULE, S., YIP, T., SHEPHERD, F., ROTSTEIN, L.E., SNIDERMAN, K.W., THEIS, E., CRAWTHORN, R.H. & RICHMOND-COX, K. (1989). Hepatocellular carcinoma: pilot trial of treatment with yttrium-90 microspheres. *Radiology*, **172**, 857-860.

INGOLD, J.A., REED, G.B., KAPLAN, H.S. & BAGSHAW, M.A. (1965). Radiation hepatitis. *Am. J. Roentgenol. Radium Ther. Nucl. Med.*, **93**, 200-208.

KAJANTI, M., RISSANEN, P., VIRKKUNEN, P., FRANSSILA, K. & MANTYLA, M. (1986). Regional intra-arterial infusion of cisplatin in primary hepatocellular carcinoma. A phase II study. *Cancer*, **58**, 2386-2388.

KOBAYASHI, H., HIDAKA, H., KAJAYA, TANOUE, H., INOUE, K., IKEDA, M., NAKAJO, M. & SHINOHARA, S. (1986). Treatment of hepatocellular carcinoma by transarterial injection of anticancer agents in iodized oil suspension or of radioactive iodized oil solution. *Acta Radiol. Diagn.*, **27**, 139-147.

LAU, W.Y. & LI, A.K.C. (1992). Therapeutic aspects of radioisotopes in hepatobiliary malignancy. *Br. J. Surg.*, **79**, 711.

LAU, W.Y., LEUNG, T.W.T., HO, S., CHAN, M., LEUNG, N.W.Y., LIN, J., METREWELI, C. & LI, A.K.C. (1994). Diagnostic pharmacoscintigraphy with technetium-99m macroaggregated albumin in the prediction of tumour to normal uptake ratio during therapy of inoperable hepatocellular carcinoma with yttrium-90 microspheres. *Br. J. Radiol.*, **67**, 136-139.

LEUNG, W.T., LAU, W.Y., HO, S.K.W., CHAN, M., NANCY, W.Y., LEUNG, N.W.Y., LIN, J., METREWELI, C., JOHNSON, P.J. & LI, A.K.C. (1994). Measuring lung shunting in hepatocellular carcinoma with intrahepatic-arterial technetium-99m macroaggregated albumin. *J. Nucl. Med.*, **35**, 70-73.

MACLINTOSH, E.L. & MINUK, G.Y. (1992). Hepatic resection in patients with cirrhosis and hepatocellular carcinoma. *Surg. Gynecol. Obstet.*, **174**, 245-254.

MALIK, S.T.A. & WRIGLEY, P.F.M. (1988). Intra-arterial hepatic chemotherapy for liver malignancy. *Br. Med. J.*, **297**, 434.

MANTRAVADI, R.V.P., SPIGOS, D.G., TAN, W.S. & FELIX, E.L. (1982). Intraarterial yttrium-90 in the treatment of hepatic malignancy. *Radiology*, **142**, 783-786.

MELIA, W.M., BULLOCK, S., JOHNSON, P.J. & WILLIAMS, R. (1983). Serum ferritin in hepatocellular carcinoma. *Cancer*, **51**, 2112-2115.

NAKANO, S., KUMADA, T., SUGIYAMA, K., WATAHIKI, H. & TAKEDA, I. (1984). Clinical significance of serum ferritin determination for hepatocellular carcinoma. *Am. J. Gastroenterol.*, 623-627.

NOVELL, J.R., HILSON, A. & HOBBS, K.E.F. (1991). Therapeutic effects of radio isotopes in hepatobiliary malignancy. *Br. J. Surg.*, **78**, 901-906.

OKUDA, K., OHTSUKI, T., OBATA, H., TOMIMATSU, M., OKAZAKI, N., HASEGAWA, H. & NAKAJIMA, Y. (1985). Natural history of hepatocellular carcinoma and prognosis in relation to treatment. *Cancer*, **56**, 918-928.

PARK, C.H., SUH, J.H., YOO, H.S., LEE, J.T., KIM, D.I. & KIM, B.S. (1987). Treatment of hepatocellular carcinoma (HCC) with radiolabelled lipiodol: a preliminary report. *Nucl. Med. Commun.*, **8**, 1075-1087.

PRINZMETAL, M., ORNITZ, Jr, E.M., SIMKIN, B. & BERGMAN, H.C. (1948). Arteriovenous anastomoses in liver, spleen and lungs. *Am. J. Physiol.*, **152**, 48-52.

SHEPHERD, F.A., ROTSEIN, L.E., HOULE, S., YIP, T.C.K., PAUL, K. & SNIDERMAN, K.W. (1992). A phase I dose escalation trial of yttrium-90 microspheres in the treatment of primary hepatocellular carcinoma. *Cancer*, **70**, 2250-2254.

SHIU, W., DEWAR, G., LEUNG, N., LEUNG, W.T., CHAN, M., TAO, M., LUI, C., CHAN, C.L., LAU, W.Y., METREWELI, C. & LI, A.K.C. (1990). Hepatocellular carcinoma in Hong Kong: clinical study on 340 cases. *Oncology*, **47**, 241-245.

YAMADA, R., SATO KAWABATA, NAKATSUKA, H., NAKAMURA, K. & TAKASHIMA, (1983). Hepatic artery embolization in 120 patients with unresectable hepatoma. *Radiology*, **148**, 397-401.

YOO, H.S., PARK, C.H., SUH, J.H., LEE, J.T., KIM, D.I., KIM, B.S. & MADSEN, M.T. (1989). Radioiodinated fatty acid esters in the management of hepatocellular carcinoma: preliminary findings. *Cancer Chemother. Pharmacol.*, **23** (Suppl.), S54-S58.